# IL-6, through p-STAT3 rather than p-STAT1, activates hepatocarcinogenesis and affects survival of hepatocellular carcinoma patients: a cohort study

**DOI:** 10.1186/s12876-015-0283-5

**Published:** 2015-04-25

**Authors:** Jung-Ta Kao, Chun-Lung Feng, Cheng-Ju Yu, Shu-Mei Tsai, Ping-Ning Hsu, Yao-Li Chen, Yi-Ying Wu

**Affiliations:** 1 School of Medicine, China Medical University, Taichung, Taiwan; 2 Graduate Institute of Clinical Medical Science, China Medical University, Taichung, Taiwan; 3 Department of Internal Medicine, Division of Hepato-Gastroenterology, China Medical University Hospital, Taichung, Taiwan; 4 Graduate Institute of Clinical Medical Science, Chang Gung University, Tao-Yuan, Taiwan; 5 Graduate Institute of Immunology, College of Medicine, National Taiwan University, Taipei, Taiwan; 6 School of Medicine, Kaohsiung Medical University, Kaohsiung, Taiwan; 7 Department of General Surgery, Changhua Christian Hospital, Changhua, Taiwan; 8 Department of Medical Laboratory Science and Biotechnology, China Medical University, No. 91, Hsueh-Shih Rd., Taichung, 404 Taiwan

**Keywords:** IL-6, p-STAT3, Chronic hepatitis, Hepatocellular carcinoma

## Abstract

**Background:**

Biologic activities of functional mediators activate downstream transducers regulating inflammation and carcinogenesis. Correlation among mediators (IL-6, IL-27, TNF-α, and VEGF) with STAT proteins at diverse clinical-pathologic stages of hepatocellular carcinoma (HCC) remains limited.

**Methods:**

Serum mediators assayed from 147 untreated HCC cases (HCC-total group) included 70 HBV-infected (HCC-HBV group), 64 HCV-infected (HCC-HCV group), and 13 without HBV-/HCV-infection (HCC-NBNC group). Another 156 non-HCC individuals comprised 54 healthy individuals (HG) and 102 chronic hepatitis patients (CH-total group) as control group. To correlate with serum mediators, 86-paired liver tissues (CH: 52 and HCC: 34 cases) served for p-STATs proteins immunostain.

**Results:**

Although four mediators (IL-6, IL-27, TNF-α, and VEGF) significantly over-expressed, IL-6 presented the strongest correlation in HCC-total versus CH-total or HG groups (HCC-total versus CH-total: P < 0.001; HCC-total versus HG: P < 0.001). Over-expressed IL-6 concentration linked with poor liver function (Albumin: r = −0.383, P < 0.001; Bilirubin: r = 0.280, P = 0.001; INR: r = 0.299, P < 0.001; AST: 0.212, P = 0.016), tumor progression (TNM system: r = 0.370; P < 0.001), clinical condition severity (BCLC system: r = 0.471; P < 0.001; terminal- versus early-stage HCC, P = 0.001; advanced- versus early-stage HCC, P = 0.007; terminal- versus intermediate- stage HCC P = 0.003; advanced- versus intermediate-stage HCC P = 0.019), and 6-month mortality (P = 0.024). Likewise, serum IL-6 (r = 0.501, P = 0.003) as compared to IL-27 (r = 0.052, P = 0.770), TNF-α (r = 0.019, P = 0.917), and VEGF (r = 0.096, P = 0.595) expression reflected positive correlation with activation of tissues p-STAT3 rather than p-STAT1.

**Conclusions:**

Serum IL-6, through p-STAT3 rather than p-STAT1 signal pathway, affected hepatic function, tumor progression, and determine HCC patient survival.

## Background

Worldwide, 711,000 new hepatocellular carcinoma (HCC) cases are diagnosed per annum, with 679,000 eventually dying [1]. Hepatitis B (HBV) and C virus (HCV) infections both contribute as leading causes [2,3]. Diagnostic and therapeutic modalities have emerged in clinical scenarios; to date, these prove inadequate due to obstacles of vascular invasion or extra-hepatic metastases [4-6]. Biochemical candidates have thus been identified by cell line or animal studies that contribute to early development and distant spread of cancer cells, but are rarely available in clinical applications [7-11]. The possibility could arise from the complicated interactions between tumor and host microenvironment in the real world. Therefore, through clinical evidence, to find the effective biomarkers and further clarify interactions with their downstream signaling targets should help greatly when evaluating actual roles in clinical settings and finally devising effective therapeutic strategies to solve this global problem.

Among well-recognized mediators, wide-ranging biological activities by IL-6, IL-27, TNF-α, and VEGF have been implicated in regulating inflammation and/or carcinogenesis [12-16]. In hepatic study, multifunctional cytokine IL-6 can stimulate hepatocyte proliferation and regeneration as well as growth modulation and tumor differentiation. High IL-6 levels might reflect more active hepatic necro-inflammation and associate with severity of disease [17-19]. Interleukin-27 (IL-27), a heterodimeric cytokine belonging to the IL-12 family, not only act on hepatocytes against viral activity but also curb tumor proliferation [20,21]. Tumor necrosis factor-alpha (TNF-α), regarded as a pro-inflammatory cytokine, is actively involved in regulation of portal hypertension and carcinogenesis [22-24]. In contrast to multifunctional activities of IL-6 and IL-27 and TNF-α in different stages of liver disease, vascular endothelial growth factor (VEGF), an essential regulator during angiogenesis rather than inflammatory process [15,25], triggers blood vessel growth for nutrition of cancer cells and affects survival in advanced HCC cases [26-28] . However, biologic function of these mediators is mediated by signaling pathways. Among them, signal transducers and activators of transcription (STATs) have been observed as essential components linking cytokine signals to transcriptional events that lead to cell proliferation, protection from apoptosis, tumorigenesis, and increased metastatic potential in various cells, including cancer [12,16,29-32]. Yet the relationship and biologic effect of functional mediators with STATs proteins is limited mostly to study of cell lines, animal models, or non-HCC patients, and is poorly understood in clinical HCC patients.

To elucidate clinical roles and relationships of IL-6, IL-27, TNF-α, and VEGF with STATs proteins at different clinical-pathological stages of HCC, we conducted this cohort study. Findings on biological mechanisms of these molecules and their interrelations with cancer might increase our understanding to create new therapeutic modalities for managing liver tumors.

## Methods

### Patients

With informed consent, 303 patients with well-characterized clinical conditions for serum mediators assay, including 147 naïve HCC patients (HCC-total group), 102 chronic hepatitis patients (CH-total group) and 54 healthy persons (HG) were enrolled in China Medical University Hospital at Taichung, Taiwan. As per clinical serological diagnoses, HCC-total patients were sub-grouped as: (1) 70 with positive HBsAg for longer than 6 months (HCC-HBV), (2) 64 with positive anti-HCV Ab for more than 6 months (HCC-HCV), and (3) 13 with negative HBsAg and anti-HCV markers (HCC-NBNC group). Hepatitis patients without HCC but with positive HBsAg or anti-HCV marker for longer than 6 months were enrolled as the chronic hepatitis (CH) group: 28 HBV- and 74 HCV-infected. Those, without HCC and negative HBsAg/ anti-HCV marker, were enrolled as a healthy group (HG). Another 86-paired liver tissues, including 34 HCC (12 HBV, 17 HCV, and 5 NBNC cases) plus 52 CH (18 HBV and 34 HCV cases) served for immunostain of STAT1 (p-STAT1) and STAT3 phosphorylation (p-STAT3).

The HCC was defined as: (1) histopathology proven by liver biopsy, or (2) image such as abdomen computerized tomography showing HCC diagnosis. Classification of HCC severity accorded with TNM and the Barcelona Clinic Liver Cancer (BCLC) staging system, as did treatment of all HCC cases. Additionally, according to median HCC survival [11] as well as rate of BCLC severity and mortality in our study, we selected 6-month mortality as cut-off point and evaluated the correlation with four mediators. Patients with (1) co-infection or super-infection (HBV or HCV); (2) prior antiviral agents like interferon or nucleoside analogues, immunomodulatory or anti-tumour agent; (3) autoimmune hepatitis or drug-induced liver disease; or (4) acute inflammation within two weeks, such as gout arthritis, were excluded. Procedures conformed to ethical standards of the responsible Committee on Human Experimentation (institutional and national) and with the 1975 Helsinki Declaration, as revised in 2008. The Institutional Review Board of China Medical University Hospital also approved this study.

### Serological virus markers and liver biochemical assays methodology

Serum HBV markers, anti-HCV antibodies, HBV DNA, and HCV RNA levels were assessed by commercial enzyme immunoassay (AxSYM, Abbott, North Chicago, IL; Abbott HCV EIA 2.0; Abbott Laboratories; Cobas Amplicor HCV Monitor 2.0; Roche Diagnostics, Branchburg, NJ). Albumin, AFP, ALT, AST, bilirubin, coagulation, and creatinine were tested by autoanalyzer (TBA-30FR, Toshiba; Tokyo, Japan).

### Estimation of serum mediators and tissue immunohistochemistry

Venous blood samples were obtained from a peripheral vein of all enrolled cases and immediately centrifuged, plasma stored at −80 °C. Quantification of IL-6, IL-27, TNF-α and VEGF by specific ELISA used commercially available kits within two weeks (IL-6, IL-27, and TNF-α used by eBioscience, San Diego, CA; VEGF used by Antigenix American, Huntington Station, NY). Results were expressed in picograms per milliliter (pg/ml), liver tissues fixed in 10% formalin and embedded in paraffin. Blocks were sectioned at 4 μm for each tissue and three pieces of each specimen stained, including one without and two with phosphorylation according to standard protocol (Cell Signaling Technology, Inc. 3 Trask Lane, Danvers, MA). The p-STAT1 and p-STAT3 immunostaining was assessed quantitatively by counting the total number of positively stained cytoplasma and nuclei of hepatocytes per 10 high-power fields (×400 magnifications) microscopically from each specimen. Positive immunostain was considered when ≧10% nuclei or cytoplasma of hepatocytes were stained [33]. The immunoreactivity expression was categorized as Level I (mean <10% nuclei or cytoplasma of hepatocytes stained, II (mean ≧10% to <25% nuclei or cytoplasma of hepatocytes stained, or III (mean ≧25% nuclei or cytoplasma of hepatocytes stained).

### Statistical analysis

Baseline data were expressed as mean ± standard deviation (Table) and mean ± standard error deviation (figures and mediators), each group of experiments repeated at least twice to confirm data. Continuous variables were assessed by Student *t*-test and Pearson correlation, data analyzed by SPSS version 17.0 for Microsoft Windows (SPSS, Chicago, IL). Two-sided *P*-value < 0.05 indicated statistical significance.

## Results

### Patients’ demographic and clinical characteristics

Table 1 shows baseline characteristics of 147 HCC, 102 hepatitis cases, and 54 healthy persons. HCC-total patients were older than those in CH-total and HG groups (65.36 ± 11.68 versus 50.20 ± 14.53 versus 42.87 ± 13.35 years respectively), which was compatible with distribution of liver diseases.

**Table 1:** **Baseline characteristics of healthy and chronic hepatitis (CH) and hepatocellular carcinoma (HCC) patients (N = 303)**

Demographics	Non-HCC group (n = 156)		HCC group (n = 147)		
	Healthy group (n = 54)	CH group (n = 102)	HCC-NBNC (n = 13)	HCC-HBV (n = 70)	HCC-HCV (n = 64)
**Age (yrs) (range)**	**42.87 ± 13.35 (19–68)**	**50.20 ± 14.53 (19–78)**	**70.38 ± 8.85 (57–85)**	**60.00 ± 11.91 (30–86)**	**69.36 ± 9.71 (47–86)**
**Sex (Male) (%)**	**33 (61.10%)**	**61 (59.8%)**	**9 (69.20%)**	**56 (80.00%)**	**37 (57.80%)**
**Cirrhosis**	**0**	**13 (12.7%)**	**9 (69.20%)**	**47 (67.10%)**	**44 (68.80%)**
**Varices/Variceal bleeding (%)**	**0/0**	**3/0**	**6/1**	**33/11**	**27/13**
**BCLC system (Early/Intermediated/Advanced/Terminal Stage)**	**−/−/−/−**	**−/−/−/−**	**0/3/7/3**	**14/10/24/22**	**13/19/17/15**
**Biochemical values**					
**Albumin (g/dL)**	**4.44 ± 0.37 (3.5-5.0)**	**4.22 ± 0.51 (2.30-5.10)**	**3.32 ± 0.56 (2.40-4.50)**	**3.33 ± 0.73 (2.00-4.90)**	**3.26 ± 0.63 (2.00-4.80)**
**Bilirubin (mg/dL)**	**0.80 ± 0.23 (0.25-1.30)**	**1.04 ± 0.50 (0.42-4.11)**	**3.42 ± 5.58 (0.44-19.84)**	**2.77 ± 3.42 (0.38-16.47)**	**1.97 ± 2.52 (0.47-14.67)**
**Creatinine (mg/dL)**	**0.83 ± 0.20 (0.49-1.51)**	**0.86 ± 0.31 (0.43-2.62)**	**1.47 ± 0.86 (0.61-3.43)**	**1.10 ± 0.99 (0.42-6.95)**	**1.50 ± 1.60 (0.47-10.66)**
**AST (IU/L)**	**21.86 ± 6.28 (14–51)**	**66.58 ± 66.16 (12–463)**	**121.10 ± 162.74 (29.00-578.00)**	**166.88 ± 428.01 (23.00-3410.00)**	**106.51 ± 106.86 (25.00-488.00)**
**ALT (IU/L)**	**20.93 ± 7.16 (10–40)**	**86.07 ± 137.34 (13–1330)**	**48.38 ± 28.49 (5.00-103.00)**	**73.11 ± 105.62 (16.00-633.00)**	**72.14 ± 60.97 (10.00-366.00)**
**INR**	**0.96 ± 0.06 (0.86-1.18)**	**1.02 ± 0.08 (0.87-1.42)**	**1.25 ± 0.41 (0.85-2.21)**	**1.27 ± 0.46 (0.89-4.50)**	**1.15 ± 0.22 (0.86-2.20)**
**Platelet (10**^**3**^**/uL)**	**245.46 ± 57.22 (138–351)**	**179.01 ± 58.24 (44–378)**	**163.62 ± 66.73 (22.00-265.00)**	**172.93 ± 91.03 (21.00-451.00)**	**124.50 ± 59.86 (18.00-323.0)**
**AFP (ng/mL)**	**2.78 ± 1.45 (1.0-8.02)**	**21.92 ± 79.29 (0.99-611.11)**	**15421.25 ± 23754.14 (2.55-54001.0)**	**11119.34 ± 20318.09 (2.05-54001.00)**	**5817.04 ± 15840.43 (1.33-54001.0)**
**Virologic values**					
**HBeAg (+) (%)**	**0**	**9 (9/28 = 32.14%)**	**0**	**7 (10%)**	**0**

### Compared to healthy group (HG), four mediators besides VEGF presented significantly in CH group

**Figure 1:**
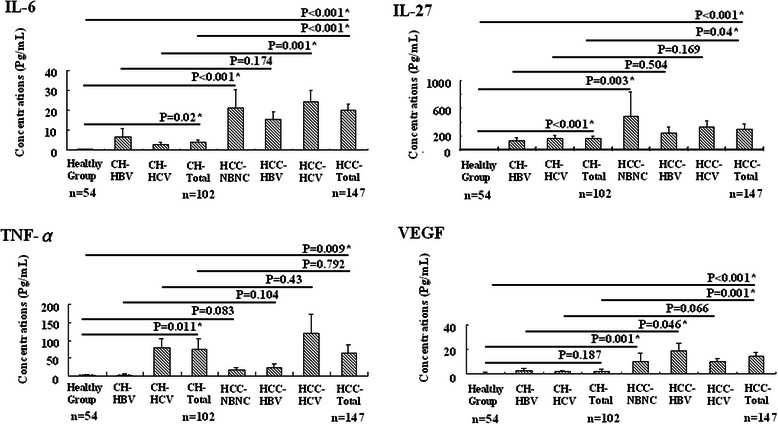
Four mediators’ expression in overall patients. Among four mediators, IL-6 presented strongest expression in HCC-total and separated HCC groups as compared to the healthy (HG) or CH-total groups, *P < 0.05 defined as statistically significant.

In HBV- or HCV-infected liver disease, IL-6, IL-27, and TNF-α presented significant expression in CH-total group than HG (3.79 ± 1.40 versus 0.46 ± 0.10 pg/ml in IL-6, P = 0.02; 164.19 ± 33.43 versus 9.83 ± 3.26 pg/ml in IL-27, P < 0.001; 75.62 ± 28.14 versus 1.76 ± 1.49 pg/ml in TNF-α, P = 0.011 respectively) (Figure 1).

### Among four mediators, over-expression of IL-6 presented the strongest correlation with HCC-total and separate HCC groups than healthy (HG) or CH-total group

While hepatocarcinogenesis, four mediators presented predominant expression in HCC-total group as compared to HG (19.70 ± 3.28 versus 0.46 ± 0.10 pg/ml in IL-6, P < 0.001; 298.46 ± 69.23 versus 9.83 ± 3.26 pg/ml in IL-27, P < 0.001; 65.40 ± 23.82 versus 1.76 ± 1.49 pg/ml in TNF-α, P = 0.009; 14.12 ± 2.91 versus 0.40 ± 0.07 pg/ml in VEGF, P < 0.001 respectively) but TNF-α failed in CH group (19.70 ± 3.28 versus 3.79 ± 1.40 pg/ml in IL-6, P < 0.001; 298.46 ± 69.23 versus 164.19 ± 33.43 pg/ml in IL-27, P = 0.040; 65.40 ± 23.82 versus 75.62 ± 28.14 pg/ml in TNF-α, P = 0.792; 14.12 ± 2.91 versus 1.79 ± 1.12 pg /ml in VEGF, P = 0.001 respectively). Furthermore, IL-6 presented the strongest expression in separate HCC groups: HCC-HBV group versus HG (15.57 ± 3.85 versus 0.46 ± 0.10 pg/ml in IL-6, P < 0.001; 240.39 ± 95.39 versus 9.83 ± 3.26 pg/ml in IL-27, P = 0.018; 24.74 ± 9.36 versus 1.76 ± 1.49 pg/ml in TNF-α, P = 0.018; 19.02 ± 5.39 versus 0.40 ± 0.07 pg/ml in VEGF, P = 0.001 respectively) and CH-HBV group (15.57 ± 3.85 versus 6.55 ± 4.31 pg/ml in IL-6, P = 0.174; 240.39 ± 95.39 versus 139.61 ± 40.51 pg/ml in IL-27, P = 0.504; 24.74 ± 9.36 versus 4.57 ± 1.49 pg/ml in TNF-α, P = 0.124; 19.02 ± 5.39 versus 2.15 ± 1.58 pg/ml in VEGF, P = 0.046 respectively); HCC-HCV group versus HG (23.96 ± 5.98 versus 0.46 ± 0.10 pg/ml in IL-6, P < 0.001; 325.98 ± 98.96 versus 9.83 ± 3.26 pg/ml in IL-27, P = 0.002; 119.77 ± 53.11 versus 1.76 ± 1.49 pg/ml in TNF-α, P = 0.030; 9.53 ± 2.77 versus 0.40 ± 0.07 pg/ml in VEGF, P = 0.002 respectively) and CH-HCV group (23.96 ± 5.98 versus 2.78 ± 1.06 pg/ml in IL-6, P = 0.001; 325.98 ± 98.96 versus 175.53 ± 44.12 pg/ml in IL-27, P = 0.169; 119.77 ± 53.11 versus 76.35 ± 28.52 pg/ml in TNF-α, P = 0.43; 9.53 ± 2.77 versus 1.65 ± 0.78 pg/ml in VEGF, P = 0.066 respectively); and HCC-NBNC group versus HG (21.14 ± 9.16 versus 0.46 ± 0.10 pg/ml in IL-6, P < 0.001; 482.75 ± 353.68 versus 9.83 ± 3.26 pg/ml in IL-27, P = 0.003; 16.05 ± 7.41 versus 1.76 ± 1.49 pg/ml in TNF-α, P = 0.083; 10.09 ± 5.96 versus 0.40 ± 0.07 pg/ml in VEGF, P = 0.001 respectively) (Figure 1).

### Among four mediators, over-expression of IL-6 correlated with deterioration of liver and tumor condition according to the BCLC staging system

**Figure 2:**
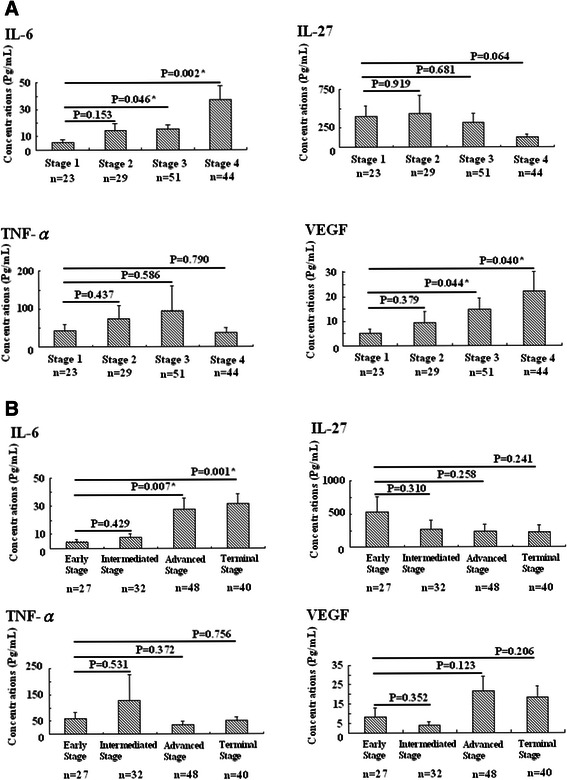
Four mediators’ expression in tumor severity. Among the four mediators, IL-6 and VEGF showed significant expressions in patients with stages 3 or 4 HCC according to the classifications of TNM staging system (Figure 2A), but only IL-6 showed a significant difference in patients with advanced or terminal-stage HCC according to classification of the BCLC staging system (Figure 2B), *P < 0.05 defined as significant.

Of four mediators, higher IL-6 level not only presented the strongest correlation with clinical factors in liver function—e.g., Albumin (r = −0.383; P < 0.001), AST (r = 0.212; P = 0.016), Bilirubin (r = 0.280; P = 0.001), INR (r = 0.299; P < 0.001) (Table 2)—but also proved significant in deteriorating patient condition, as per the BCLC scoring system (terminal- versus early-stage HCC: 31.28 ± 7.14 versus 4.87 ± 1.66 pg/ml, P = 0.001; terminal- versus intermediate-stage HCC: 31.28 ± 7.14 versus 7.61 ± 2.84 pg/ml, P = 0.003; advanced- versus early-stage HCC: 27.70 ± 7.83 versus 4.87 ± 1.66 pg/ml, P = 0.007; advanced- versus intermediate-stage HCC 27.70 ± 7.83 versus 7.61 ± 2.84 pg/ml, P = 0.019). Conversely, IL-27, TNF-α, and VEGF could not present significance in each stage (Figure 2).

**Table 2:** **Correlations between IL-6, IL-27, TNF-α, and VEGF with biochemical data in HCC patients**

	IL-6		IL-27		TNF-α		VEGF	
	γ	*P*	γ	*P*	γ	*P*	Γ	*P*
**Age (years)**	**0.137**	**0.106**	**−0.016**	**0.855**	**−0.013**	**0.882**	**−0.078**	**0.363**
**Sex (F/M)**	**0.057**	**0.504**	**−0.052**	**0.538**	**−0.215**	0.011*	**0.023**	**0.788**
**Varices (−/+)**	**0.155**	**0.067**	**0.024**	**0.775**	**0.058**	**0.495**	**0.152**	**0.075**
**Variceal bleeding (−/+)**	**0.135**	**0.112**	**0.042**	**0.625**	**0.215**	0.011*	**0.154**	**0.071**
**Albumin (g/dL)**	**−0.383**	<0.001*	**−0.098**	**0.253**	**−0.031**	**0.717**	**−0.175**	0.041*
**Bilirubin (mg/dL)**	**0.280**	0.001*	**0.046**	**0.590**	**0.161**	**0.060**	**−0.112**	**0.175**
**Creatinine (mg/dL)**	**0.181**	0.032*	**−0.037**	**0.663**	**0.022**	**0.792**	**−0.074**	**0.391**
**AST (IU/L)**	**0.212**	0.016*	**0.112**	**0.208**	**0.092**	**0.300**	**0.020**	**0.819**
**ALT (IU/L)**	**−0.087**	**0.305**	**0.083**	**0.329**	**0.087**	**0.305**	**−0.084**	**0.325**
**INR**	**0.299**	<0.001*	**−0.064**	**0.460**	**−0.095**	**0.269**	**0.160**	**0.063**
**Platelet (10** ^**3**^ **/uL)**	**0.036**	**0.671**	**0.043**	**0.619**	**0.016**	**0.857**	**0.002**	**0.985**
**AFP (ng/mL)**	**0.261**	0.002*	**0.002**	**0.977**	**0.120**	**0.157**	**0.123**	**0.152**
**TNM Staging**	**0.370**	<0.001*	**−0.109**	**0.200**	**−0.052**	**0.539**	**0.059**	**0.491**
**BCLC Staging**	**0.471**	<0.001*	**−0.028**	**0.739**	**0.035**	**0.682**	**0.158**	**0.065**

(N = 147) *p < 0.05 defined as significant

### Among four mediators, IL-6 over-expression predicted obviously 6-month mortality

**Figure 3:**
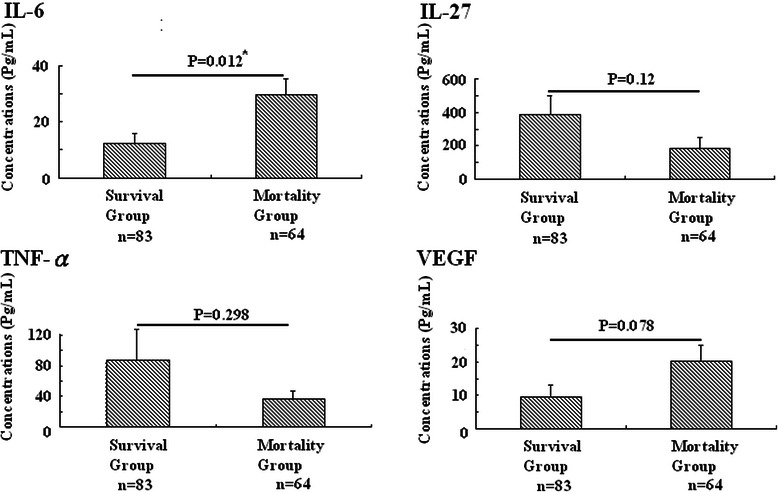
Comparison of four mediators’ expression with patient’s mortality. Among four mediators, IL-6 presented strongest correlation with 6-month mortality in HCC patients (Figure 3), *P < 0.05 defined as significant.

Compatible with the above, over-expression of IL-6 correlated with mortality (<6-month versus ≥ 6-month survival: 29.66 ± 5.86 versus 12.23 ± 3.51 pg/ml, P = 0.012, respectively) while portending 6-month mortality in HCC-total cases (Table 3). By contrast, elevated IL-27, TNF-α, or VEGF presented no significant correlation with 6-month mortality (Figure 3).

**Table 3:** **Univariate cox regression model of baseline characteristics and 6-month mortality in hepatocellular carcinoma (HCC) patients**

Variable	Parameter estimate	SE of estimate	Risk ratio (95%CI)	*P*value
**Demographics**				
**Age (yrs)**	**0.011**	**0.016**	**1.011(0.981-1.043)**	**0.465**
**Gender (F/M)**	**0.532**	**0.392**	**1.702(0.790-3.668)**	**0.174**
**Varices (−/+)**	**1.490**	**0.381**	**4.438(2.101-9.373)**	<0.001*
**Variceal rupture (−/+)**	**1.496**	**0.493**	**4.463(1.697-11.739)**	0.002*
**IL-6 (pg/mL)**	**0.015**	**0.007**	**1.015(1.002-1.028)**	0.024*
**IL-27 (pg/mL)**	**0.000**	**0.000**	**1.000(0.999-1.000)**	**0.204**
**TNF-α (pg/mL)**	**−0.001**	**0.001**	**0.999(0.996-1.002)**	**0.401**
**VEGF (pg/mL)**	**0.034**	**0.013**	**1.034(1.009-1.060)**	0.007*
**AST (IU/L)**	**0.008**	**0.003**	**1.008(1.003-1.013)**	0.003*
**ALT (IU/L)**	**0.002**	**0.002**	**1.002(0.998-1.007)**	**0.337**
**Albumin (g/dL)**	**−2.413**	**0.444**	**0.090(0.038-0.214)**	<0.001*
**Bilirubin (mg/dL)**	**0.455**	**0.132**	**1.576(1.216-2.042)**	0.001*
**Cr (mg/dL)**	**−0.014**	**0.132**	**0.986(0.761-1.278**	**0.914**
**INR**	**5.763**	**1.359**	**318.386(22.17-4572.329)**	<0.001*
**Platelet (10**^**3**^**/uL)**	**0.001**	**0.002**	**1.001(0.997-1.006)**	**0.576**
**AFP (ng/mL)**	**0.000**	**0.000**	**1.000(1.000-1.000)**	0.001*
**Cirrhosis (−/+)**	**1.131**	**0.422**	**3.10(1.357-7.083)**	0.007*
**BCLC staging**	**2.394**	**0.411**	**10.958(4.897-24.522)**	<0.001*

(N = 147) *p < 0.05 defined as statistically significant.

### Liver preservation (Child-Pugh Classification) rather than presence or absence of cirrhosis presented high correlation with IL-6 over-expression

There was no link between four mediators with presence or not of cirrhosis (19.80 ± 4.03 versus 19.50 ± 5.70 pg/ml in IL-6, P = 0.966; 299.30 ± 79.70 versus 296.67 ± 135.87 pg/ml, P = 0.986 in IL-27; 74.13 ± 34.09 versus 46.98 ± 17.98 pg/ml in TNF-α, P = 0.596; 12.92 ± 2.80 versus 16.60 ± 6.82 pg/ml in VEGF, P = 0.555 respectively). However, over-expression of IL-6 strongly correlated with Child-Pugh Classification and 6-month mortality in HCC-total (IL-6: r = 0.365, P = 0.002; IL-27: r = 0.119, P = 0.325; TNF-α: r = 0.059, P = 0.628; VEGF: r = 0.111, P = 0.363 respectively; IL-6: r = 0.488, P < 0.001; IL-27: r = −0.109, P = 0.244; TNF-α: r = 0.038, P = 0.684; VEGF: r = 0.214, P = 0.022 respectively) as well as cirrhotic HCC cases (IL-6: r = 0.376, P = 0.002; IL-27: r = 0.183, P = 0.139; TNF-α: r = 0.143, P = 0.247; VEGF: r = 0.089, P = 0.480 respectively; IL-6: r = 0.577, P < 0.001; IL-27: r = −0.036, P = 0.750; TNF-α: r = 0.085, P = 0.451; VEGF: r = 0.213, P = 0.059 respectively).

### Rather than tissue p-STAT1, tissue p-STAT3 showed predominant immunostain rate and correlated with serum IL-6 expressions among four mediators in HCC

**Figure 4:**
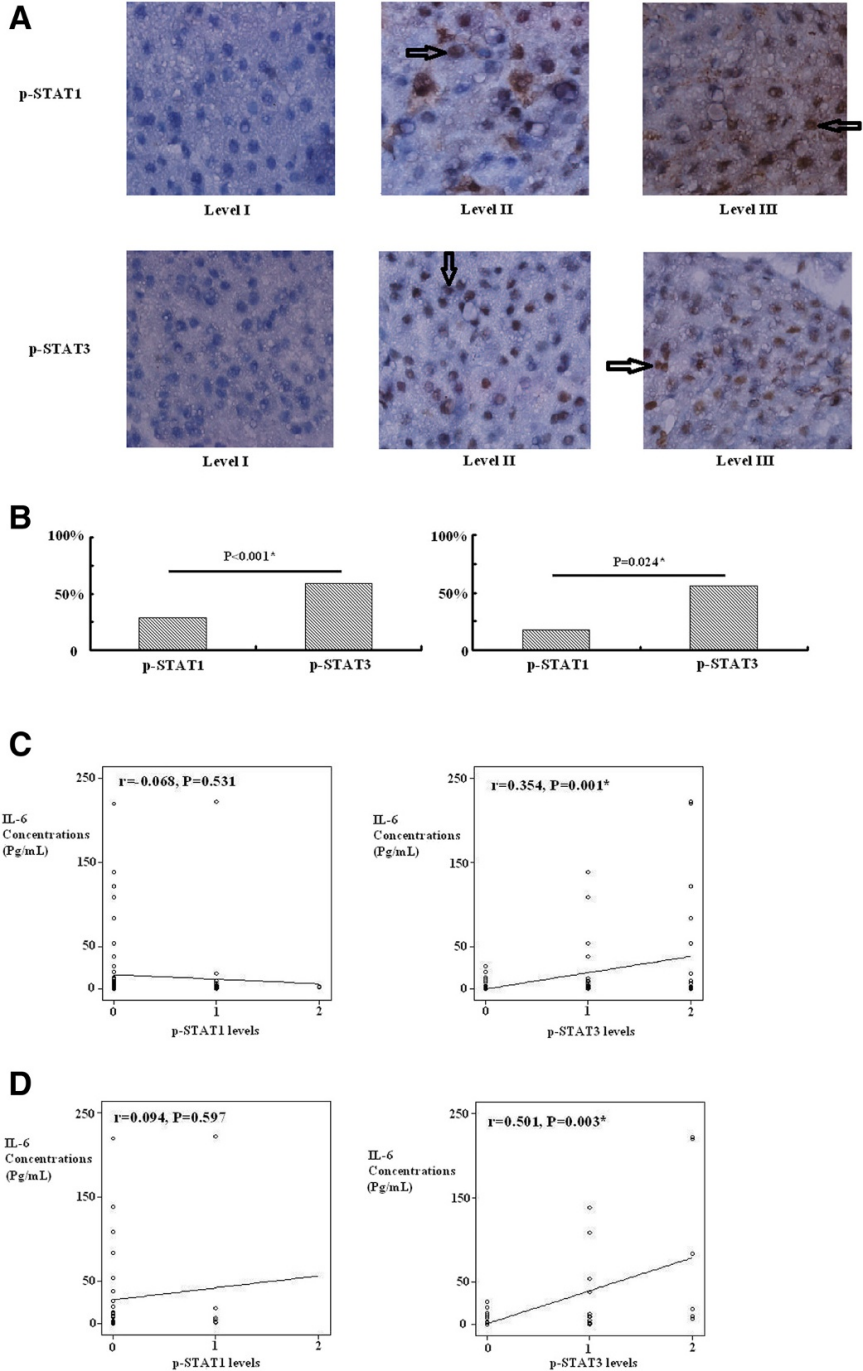
Correlation between p-STAT1 and p-STAT3 with IL-6 expression. Immunostain expression of p-STAT1 and p-STAT3 in three identical HCC tissues. Immunoreactivity exhibited in nuclei or cytoplasm of hepatocyte was designated Level I (<10%), II (≧10% to ≦25%), or III (≧25%) (×400 magnifications) (brown cell as arrow shows positive immunostain) (Figure 4A). Tissue p-STAT3 presented a predominant immunostain rate (≧10% immunoreactivities) unlike p-STAT1 in all specimens (Figure 4B left) and HCC specimens (Figure 4B right). Rising immunoreactivity of p-STAT3 versus p-STAT1 positively correlated with over-expression of serum IL-6 rather than IL-27 and TNF-α and VEGF in all specimens (Figure 4C) and HCC specimens (Figure 4D) (relationships of IL-27 and TNF-α and VEGF with p-STAT1 and p-STAT3 shown in Results), *P < 0.05 defined as significant.

To elucidate clinical relationship between tissue p-STAT proteins and serum mediators, we analyzed immunohistochemical expression of p-STAT1 and p-STAT3 in 86 biopsies (52 CH and 34 HCC cases). Tissue p-STAT3 presented a predominant immunostain rate (≧10% immunostaining of hepatocytes) as compared to that of p-STAT1 in all liver (51/86 versus 25/86, P < 0.001) and CH (32/52 versus 19/52, P = 0.003) and HCC specimens (19/34 versus 6/34, P = 0.024) (Figures 4A, B). Notably, rising immunoreactivity levels of p-STAT3 instead of p-STAT1 presented positive correlation with over-expression of serum IL-6 rather than IL-27, TNF-α, and VEGF not only in overall (p-STAT3: r = 0.354, P = 0.001; r = 0.116, P = 0.289; r = 0.125, P = 0.253; r = 0.175, P = 0.109 respectively) (p-STAT1: r = −0.068, P = 0.531; r = −0.085, P = 0.438; r = 0.013, P = 0.907; r = −0.057, P = 0.606 respectively) but also in HCC cases (p-STAT3: r = 0.501, P = 0.003; r = 0.052, P = 0.770; r = 0.019, P = 0.917; r = 0.096, P = 0.595 respectively) (p-STAT1: r = 0.094, P = 0.597; r = −0.197, P = 0.264; r = −0.093, P = 0.599; r = −0.118, P = 0.511 respectively) (Figures 4C, D).

## Discussion

Biologic activities of functional mediators activating their downstream special transducer are prominent in regulation of inflammation and carcinogenesis. Still, the evidence of biologic mechanisms and their interrelations with cancer between functional IL-6, IL-27, TNF-α, and VEGF with STAT protein is mostly limited to cell line or animal models or non-HCC patients, and poorly understood in liver disease, especially in diverse clinical-pathologic stages of HCC.

The stimulation particularly by hepatitis B or C infection can activate host immune mechanisms to drive serum functional mediators that reflect inflammatory processes and modulate liver regeneration. Indeed, our study only found expressions of serum IL-6, IL-27, and TNF-α rather than VEGF obvious in CH-total rather than HG groups (Figure 1), which concurred with previous study: IL-6, IL-27, or TNF-α playing an inflammatory role in regulating hepatocyte proliferation and regeneration, and VEGF playing a major role in pathogenesis of liver cancer [17,18,20,22,23,26,28].

In addition to playing potential inflammation regulators in IL-6, IL-27, and TNF-α, our study found these mediators with VEGF expressed strongly in HCC-total or separate HCC as compared to CH or HG cases (Figure 1), which was compatible with prior studies: IL-6, IL-27, and TNF-α could, like VEGF, play a pivotal role in carcinogenesis [12,16,24,26,28]. However, correlating their expression with tumor severity (TNM staging system), only IL-6 and VEGF presented a positive trend with tumor progression (Figure 2A). In clinical settings, therapeutic standard and survival prediction of HCC cases closely relates to levels of the BCLC staging system, combining with tumor severity, liver function, and performance status. We found IL-6 instead of VEGF expressed obvious correlation not only with tumor severity but also with deteriorating liver preservation (Figure 2B). This finding was also supported by parameters associated with impaired liver function: AST (r = 0.212; P = 0.016), albumin (r = −0.383; P < 0.001), bilirubin (r = 0.280; P = 0.001), INR (r = 0.299; P < 0.001), AFP (r = 0.261; P = 0.002), and creatinine (r = 0.181; P = 0.032) significantly correlated with patient’s survival when IL-6 was over-expressed rather than other mediators (Figure 3, Tables 2 and 3). Cirrhosis plays an important role in pathogenesis of liver cancer and patient survival, which also concurred with our result (Table 3). Yet we observed presence or absence of cirrhosis in HCC patients not reflecting expression of IL-6, IL-27, TNF-α, and VEGF. This might be complicated and contribute to the liver situation. While correlating among IL-6, IL-27, TNF-α, and VEGF with Child-Pugh Classification and 6-month mortality with presence or not of cirrhosis, only IL-6 over-expression strongly correlated with preservation of liver function and 6-month mortality in HCC-total (r = 0.365; P = 0.002, r = 0.488; P < 0.001 respectively) or cirrhotic HCC (r = 0.376; P = 0.002, r = 0.577; P < 0.001 respectively). Findings can explain this discordance. It yields clinical evidence of IL-6 linked with disease progression as compared with IL-27, TNF-α, and VEGF in HCC cases.

Several signaling pathways mediating biologic effects of these mediators, and STAT signaling pathway plays an essential component linking cytokine signals to transcription, inducing cell proliferation, protection from apoptosis, tumorigenesis, and higher metastatic potential in diverse cells [12,16,29-32]. However, clinical correlation between IL-6, IL-27, TNF-α, and VEGF with different clinical-pathologic stages of HCC remains limited and warrants further clarification. Both STAT1 and STAT3 proteins have been implicated as essential components linking cytokines signals to transcriptional events in pathogenesis of liver disease [12,16,29]. To correlate STAT1 and STAT3 with their mediators, we examined CH and HCC tissues to probe immunostain expressions. Tissues with p-STAT3 expressed a higher immunostain rate overall than p-STAT1 in all liver (P < 0.001), or separate CH (P = 0.003) and HCC specimens (P = 0.024) (Figures 4A, B), while rising immunoreactivity level of p-STAT3 reflected significant correlation with IL-6 expression as compared to IL-27, TNF-α, and VEGF expression in all patients (P = 0.001; P = 0.289; P = 0.253; P = 0.109 respectively) or HCC (P = 0.003; P = 0.770; P = 0.917; P = 0.595 respectively) (Figures 4C, D). This confirmed a mechanism: IL-6 major through p-STAT3 rather than p-STAT1 pathway affecting severity of inflammation and carcinogenesis in liver disease, particularly in HCC patients [12,16,29]. This finding was also concurred with previous study that STAT3 activated by exogenous IL-6 cytokine played a functional role in cholangiocarcinoma development and associated with patient’s survival, which belonged to hepatobiliary malignancies [33].

Our study failed to model all participants’ liver tissue, this limit based on ethical and safety considerations: HCC patients with decompensated liver disease usually have high hemorrhagic risk. While VEGF presented strong correlation with 6-month mortality (Table 3), it did not reflect as readily as IL-6, which might indicate need for activation via other signal cascades like JAK/STAT pathway in carcinogenesis [5,26,34]. Fluctuating concentration of serum IL-6, IL-27, TNF-α, and VEGF in the host might be argued, but these plasma molecules assessed were not affected by time between blood sampling and centrifuge, according to prior study [35].

## Conclusions

Take together, our findings clearly demonstrate serum IL-6 rather than IL-27, TNF-α, and VEGF playing a definite role in liver deterioration and tumor progression, as well as further affecting HCC patient survival. The mechanism of IL-6 biologic activity is chiefly through activation of p-STAT3 instead of p-STAT1 protein in the real world. From functional identification of IL-6/p-STAT3 pathways, we believe ELISA detection of circulating IL-6 and immunostain of tissue p-STAT3 as biomarker combined with current clinical biochemical data or images can provide clinicians with useful references for prognosis. Such an attractive immunotherapeutic strategy would reduce or prevent mortality in the future.
